# Distinct gene expression patterns correlate with developmental and functional traits of iNKT subsets

**DOI:** 10.1038/ncomms13116

**Published:** 2016-10-10

**Authors:** Hristo Georgiev, Inga Ravens, Charaf Benarafa, Reinhold Förster, Günter Bernhardt

**Affiliations:** 1 Institute of Immunology, Hannover Medical School, Carl Neuberg Street 1, Hannover D-30625, Germany; 2 Theodor Kocher Institute, University of Bern, Freisestrasse 1, Bern CH-3012, Switzerland

## Abstract

Invariant natural killer T (iNKT) cells comprise a subpopulation of innate lymphocytes developing in thymus. A new model proposes subdividing murine iNKT cells into iNKT1, 2 and 17 cells. Here, we use transcriptome analyses of iNKT1, 2 and 17 subsets isolated from BALB/c and C57BL/6 thymi to identify candidate genes that may affect iNKT cell development, migration or function. We show that Fcɛr1γ is involved in generation of iNKT1 cells and that SerpinB1 modulates frequency of iNKT17 cells. Moreover, a considerable proportion of iNKT17 cells express IL-4 and IL-17 simultaneously. The results presented not only validate the usefulness of the iNKT1/2/17-concept but also provide new insights into iNKT cell biology.

## Abstract (web summary)

A recent advance in invariant natural killer T cell (iNKT) cell biology is their classification into iNKT1, iNKT2 and iNKT17 subsets. Here the authors provide a transcriptomic analysis of these thymic subsets from Balb/c and C57Bl/6 mice that supports and extends the categorization.

## Graphical abstract

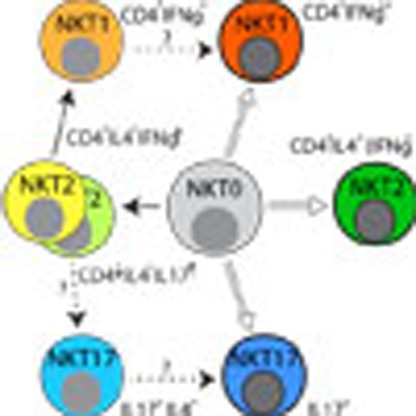

Natural killer T cells can be grouped into several subtypes of which the type I invariant natural killer T (iNKT) cells are most vigorously investigated^1^. This is due to the ease of detecting them using CD1d-tetramers loaded with α-galactosylceramide (α-galcer) or a glycolipid derived from it. Given the highly restricted set of TCRα,β-chains expressed (Vα14Jα18-Vβ2,7,8.1/2/3 in mouse) coining the name invariant, iNKT cells are involved in a surprisingly wide range of immune relevant processes such as activating NK or B cell^2^,^3^ or biasing T cell responses and activities of dendritic cells (DC)^4^,^5^. Consequently, iNKT cells can influence the outcome of various diseases ranging from bacterial or viral infection^6^,^7^ and cancer^8^ to autoimmune and allergy syndromes^9^,^10^. These findings fostered interest in this highly specialized T cell type that comes into existence in the thymus when T cells pass through the double positive stage of their differentiation^11^. iNKT cells differ from regular naïve T cells not only in the limited set of T cell receptors (TCR) expressed, but also in their quasi antigen experienced status that enables immediate reaction to TCR-mediated or cytokine-induced stimuli by secreting a variety of cytokines^12^,^13^,^14^. In addition, in contrast to naive T cells, iNKT cells can leave the thymus as immature cells and complete differentiation in the periphery^15^,^16^ with minimal recirculation^17^. Moreover, iNKT cells express a variety of homing receptors licensing them to migrate to lymphoid but also non-lymphoid organs, including skin, liver and lung^18^.

Much of our insights regarding murine iNKT cells were derived from experimentation in C57Bl/6 mice, the strain that also served to establish the classical model subdividing iNKT cells according to their developmental stages, S0–S3 (ref. 19). This classification rests in part on the marker NK1.1 defining the iNKT cell stages as follows: S0 (CD24^+^CD44^lo^NK1.1^lo^); S1 (CD24^lo^CD44^lo^NK1.1^lo^); S2 (CD24^lo^CD44^hi^NK1.1^lo^); S3 (CD24^lo^CD44^hi^NK1.1^hi^)^15^,^16^. Differentiating iNKT cells switch from a predominant IL-4 secretion to predominant IFNγ production, a process termed TH2 to TH1 conversion^15^. However, NK1.1 is not expressed by many other mouse strains, including BALB/c mice, thereby impeding comparability of iNKT subtypes. Moreover, it was difficult to integrate IL-17 producing iNKT cells, discovered later, into the established concept^20^. iNKT cell differentiation is governed by key transcription factors PLZF, TBET, GATA3, THPOK and RORγt^21^,^22^ that serve as markers to define murine iNKT subtypes^23^. A subdivision of iNKT cells identified by expression of PLZF, TBET and RORγt matches well with the secretion of key cytokines IFNγ, IL-4 and IL-17, respectively^11^,^20^,^23^. Following the T_H_1/2/17-paradigm, iNKT cells can thus be defined as PLZF^lo^T-bet^+^RORγt^−^ iNKT1 (IFNγ^+^), PLZF^hi^T-bet^−^RORγt^−^ iNKT2 (IL-4^+^) and PLZF^int^T-bet^−^RORγt^+^ iNKT17 (IL-17^+^) cells providing a solid platform to also discriminate iNKT cells by their functional qualities^1^,^11^. Comparing the classical concept (S0–S3) with the recently described classification (iNKT1/2/17) it is evident that, neglecting sharp borders, iNKT2 cells correspond to more immature iNKT cells whereas iNKT1 cells represent terminally differentiated cells. However, iNKT2 cells actively secreting IL-4 cannot give rise to the more mature iNKT1 cells^23^, raising doubts of a straight-forward developmental programme executed by differentiating iNKT cells. An alternative differentiation pathway is that iNKT1, 2 and 17 cells develop directly from a common precursor. Despite these unresolved issues, the iNKT1/2/17-concept has gained quick acceptance.

Although transcriptome analyses of iNKT cells have been published^24^,^25^,^26^, only one study has provided new insights into iNKT cell function and development based on the iNKT1/2/17-classification^27^. In the study presented here, we used a simple gating strategy to investigate the transcriptomes of iNKT1, 2 and 17 cells from thymus of BALB/c and C57BL/6 mice. The results confirmed that a subdivision into iNKT1, 2 and 17 cells is suitable to characterize iNKT cells independent of the strain but also revealed candidate genes that may explain strain dependent variations in iNKT subset composition reported earlier^23^. We identify many genes that are expressed in a subtype specific fashion in both strains and by investigating corresponding mutant mice, we show that Fcɛr1γ and serpinB1 are involved in generating wild type (WT)-like iNKT subset compositions. Furthermore, we investigate the importance of receptors known to be important for migration of iNKT cells. Notably, we observe that iNKT17 cells express IL-4 to a substantial extent indicating a hitherto unrecognized heterogeneity in this subpopulation. Along these lines, CD4^−/lo^ iNKT1 cells differ from CD4^+^iNKT1 cells with respect to their NK-like phenotype. These observations indicate that not only iNKT2 but also iNKT1 and iNKT17 subsets are composed of at least two subpopulations. Nevertheless, in their entity the data broadly and impressively support the theoretical fundament of the iNKT1/2/17-concept.

## Results

### Transcriptome analysis of thymic iNKT subsets

**Figure 1:**
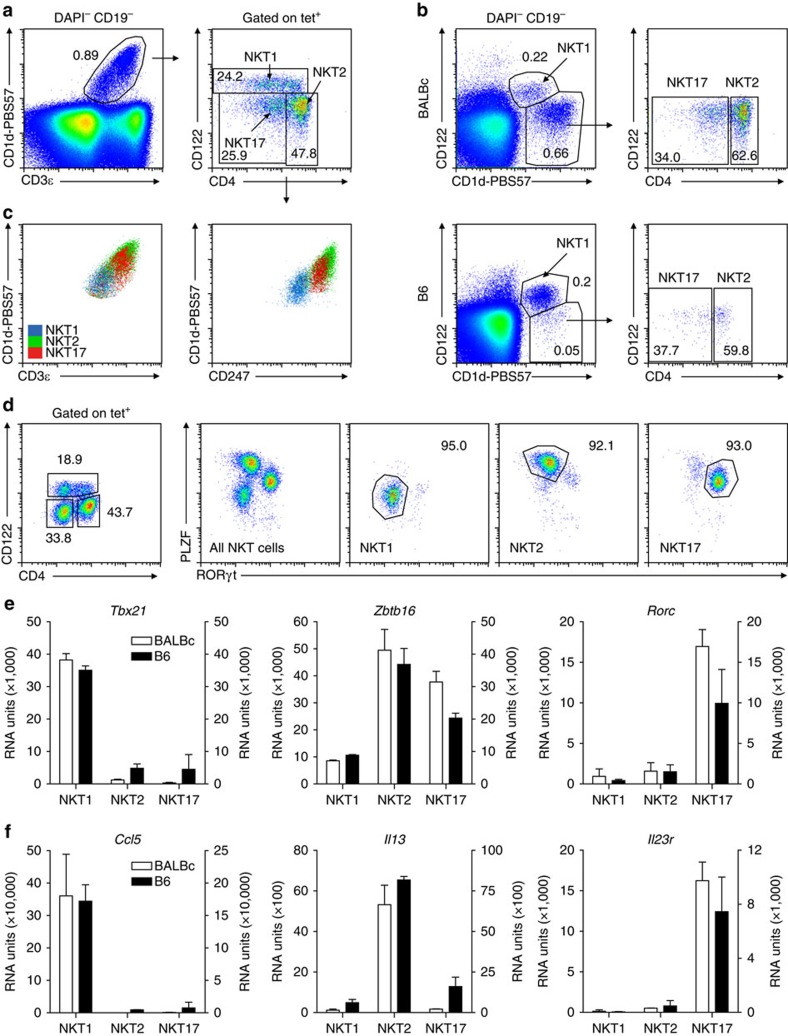
Discrimination of iNKT subsets using various markers. (**a**) CD4 and CD122 expression discriminates iNKT1, 2 and 17 populations among DAPI^−^CD19^−^ thymocytes as indicated by arrows. (**b**) Alternative representation of iNKT populations of thymus of BALB/c (upper panel) and of B6 (lower panel) using markers CD3ɛ, CD1d-tetramer, CD4 and CD122 as in **a**. The numbers in **a**,**b** indicate the frequencies of the cells in the corresponding gates. (**c**) iNKT1, 2 and 17 cells gated as in **a** expressed different levels of CD3ɛ (left panel, surface stain) or CD247 (CD3ζ, right panel, permeabilized cells). (**d**) iNKT subpopulations defined by CD4/CD122 expression were monitored for their expression of PLZF and RORγt. Gates shown in left panel were applied to define iNKT subsets and to test their purity with regard to expression of PLZF and RORγt (three panels to the right). Numbers reflect percentage of cells in the encircled areas. (**e**,**f**) mRNA expression profiles resulting from chip-based transcriptome analysis as described in the text. RU represents an arbitrary definition of expression strength. Open bars: values for BALB/c cells, scale to the left; black bars: values for B6, scale to the right. Shown are means±s.d. Data are representative of at least 5 (**a**), 5 (**b**), 5 (**c**, left panel), 2 (**c**, right panel) and 2 (**d**) experiments. Data shown in **e**,**f** are from two independently performed transcriptome analyses each, BALB/c and B6. Shown are mean±s.d. (*n*=2).

**Figure 2:**
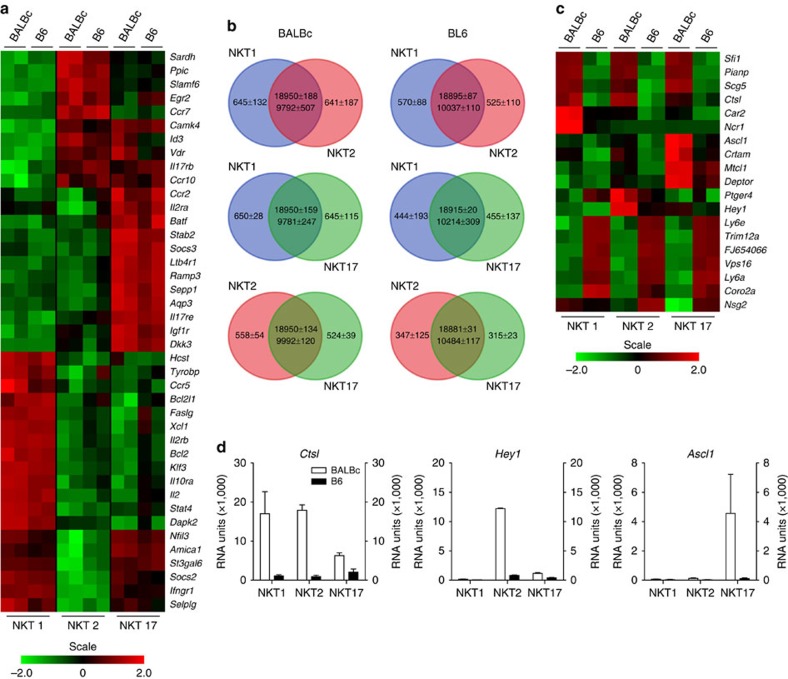
Evaluation and summary of transcriptome data. (**a**,**c**) Heat maps representing expression of the genes listed to the right. Data source as in Fig. 1e,f (*n*=2). (**b**) Venn diagrams resulting from pairwise comparisons of iNKT subsets. To generate the Venn diagrams, expression levels below 500 RU were considered negligible. Therefore, in the overlapping area the upper number encompasses all genes that were expressed at a level <500 RU in both subtypes whereas the lower number comprises all genes that were found to be expressed at a level >500 RU in at least one of the subsets but where the difference in expression was less than twofold. The numbers given in the unique fields of each diagram contain all genes that were expressed at a level >500 RU with a difference of more than twofold compared with expression in the partner subset. These genes were considered to be expressed in a iNKT subtype specific fashion. Values represent mean±s.d. of two independent experiments (*n*=2). (**d**) mRNA expression profiles resulting from transcriptome analyses as depicted in Fig. 1e,f (mean±s.d., *n*=2). Shown are three selected genes that exhibited a mouse strain specific pattern: *Ctsl* (cathepsin L), *Hey1* (HESR1) and *Ascl1* (MASH1).

iNKT1, 2 and 17 cells are usually identified by the expression pattern regarding transcription factors that requires permeabilization of the cells. However, Lee *et al*.^23^ showed that a combination of antibodies recognizing CD122, CD4 and CD27 is also suitable to identify iNKT subtypes among tetramer (tet)^+^ cells. We noted that already the use of CD122 and CD4 results in an adequate separation of tet^+^ gated thymocytes into iNKT1, 2 and 17 cells (Fig. 1a,b). This is proven by their homogeneous expression of PLZF and TBET (Fig. 1d). Next, we sorted iNKT cells of thymus of BALB/c and C57BL/6 (B6) mice into iNKT1, 2 and 17 cells according to the gating strategy shown in Fig. 1b. RNA was prepared and samples processed for hybridization of Agilent chips as described in Methods. We performed two completely independent experiments each for BALB/c and B6 (GEO accession number GSE69120). Except for the heat maps, we display the results obtained as intensity data (RNA units (RU)) in bar diagrams as means±s.d. to illustrate that both analyses, for BALB/c as well as for B6, yielded consistent findings. Although this suggests a high degree of reliability, it should be noted that the low number of replicates, *n*=2, still represents an element of uncertainty. mRNA data reflecting expression of TBET (*Tbx21*), PLZF (*Zbtb16*), and RORγ,γt (*Rorc*) matched expectations (Fig. 1e) and expression patterns of *Ccl5*, *Il13* as well as *Il23r* are shown as further examples to demonstrate that the cell sorts were clean enough allowing reliable characterization of iNKT subsets based in their transcriptional profiles (Fig. 1f). CCL5 is produced by activated NK cells and among iNKT cells, iNKT1 cells resemble most NK cells (see later). IL-13 is expressed by iNKT2 but not iNKT1 and iNKT17 cells^28^ and the receptor for IL-23 is critically involved in IL-17 expression by iNKT17 cells^22^. The heat map in Fig. 2a depicts a series of genes displaying subtype specific expression. Global analysis of the chip data reveals that within given margins (legend to Fig. 2) nearby 20,000 genes were low to not expressed in pairwise comparisons of iNKT subtypes, whereas ∼10,000 genes were expressed but did not differ substantially in expression strength (less than twofold, Fig. 2b). Approximately 300–600 transcripts were classified unique. Even though the vast majority of genes exhibit an expression pattern identical or highly similar between cells of BALB/c and B6 origin, a noticeable set of genes differ in their expression when comparing iNKT subsets of these two mouse strains (Fig. 2c,d; Supplementary Table 1).

### Expression of the TCR complex characterizes iNKT subtypes

**Figure 3:**
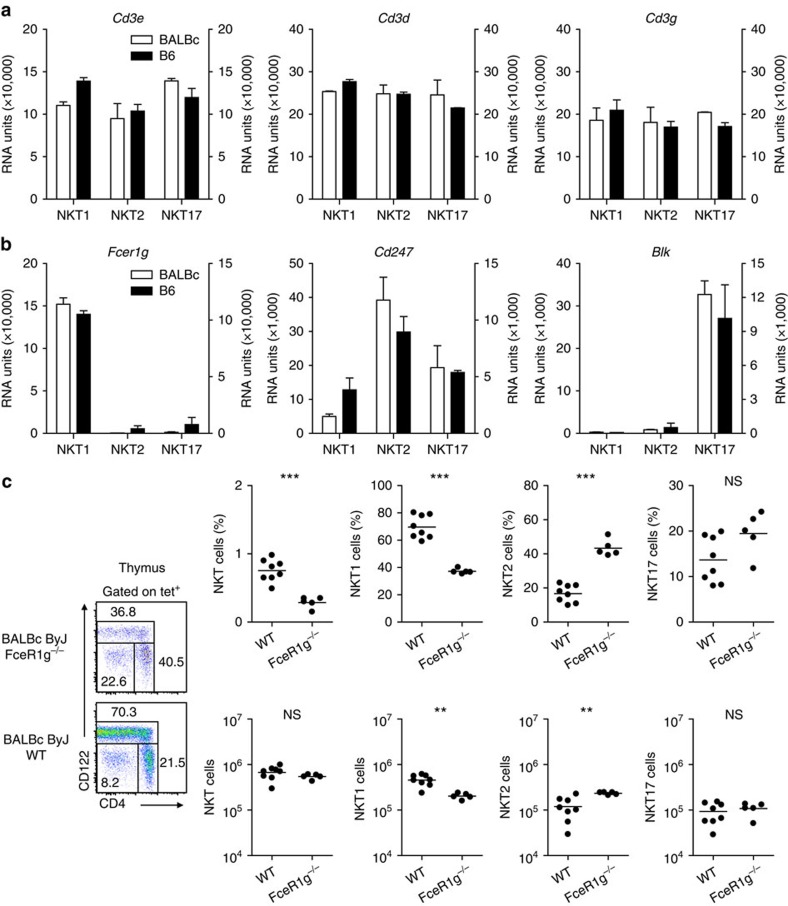
Expression of genes modulating TCR-mediated signalling. (**a**,**b**) Expression of the indicated genes in the iNKT subsets depicted as in Fig. 1e,f (mean±s.d., *n*=2). (**c**) iNKT cells (DAPI^−^B220^−^tet^+^) were gated as shown in the left panels to determine iNKT subtype composition (panels to the right). The data shown in the left two panels of **c** are representative of at least five individually stained thymi. Data shown in the right panels of **c** were collected from two independent experiments. Each dot represents one animal (WT: *n*=8, ko: *n*=5). Unpaired two-tailed *t*-test was performed. NS, not significant (*P*>0.05), **P<0.01 ***P<0.001.

Interestingly, apart from CD4 (ref. 29), marked iNKT subtype specific variations exist in the expression of several genes implicated in TCR signalling. mRNAs coding for CD3ɛ but also CD3δ and CD3γ are highly and equally well expressed among all iNKT subsets (Fig. 3a). However, when dissecting iNKT subpopulations as shown in Fig. 1a (right panel), it is clear that their CD3ɛ/TCR-expression is different in strength depending on the iNKT subtype (Fig. 1c, left panel). Therefore, in case of CD3ɛ the amount of mRNA expressed doesn't correspond to the level of protein detected on the surface of the cells. In contrast, the mRNA signal intensity for the CD3ζ chain (CD247) (Fig. 3b) varies greatly depending on the iNKT subtype and its expression levels mirrors the amount of TCR or CD3ɛ detected on the surface of the corresponding iNKT cell subtypes (Fig. 1c, right panel). This observation suggests that the CD3ζ chain is an important determinant governing levels of TCR surface expression^30^ thereby influencing the strength of the TCR signal. Interestingly, the low expression of CD3ζ in iNKT1 cells coincides with a strong mRNA signal for the FcɛR1γ chain (Fig. 3b) that is part of the high-affinity receptor for IgE and can also pair with the CD3ζ chain in the NCR1 signalling adaptor^31^ as well as the TCR complex^32^,^33^. This modified complex is likely to trigger altered TCR signalling^34^. Indeed, in thymus of BALB/cByJ mice deficient for the FcɛR1γ chain iNKT cells are reduced in frequency but not number as compared with WT (Fig. 3c, please note that the strain BALB/cByJ differs from the commonly used strain BALB/c). More precisely, iNKT1 cell frequency and number are lower compared with WT controls whereas those of iNTK2 increase. In periphery, differences regarding composition of iNKT subtypes are not evident (spleen) or much less pronounced (liver) when comparing knock-out and WT animals although overall frequency of iNKT cells is decreased in spleen (Supplementary Fig. 1a,b). Most likely, not only does this phenomenon reflects the well-known capacity of peripheral iNKT cells to propagate and differentiate but also indicates that the maturation pathways or kinetics in thymus differ from those in periphery^35^. These results were confirmed when iNKT subsets were identified based on their expression of PLZF and RORγt (Supplementary Fig. 1c,d). Moreover, the chip data reveal a high and very specific expression of the B lymphoid tyrosine kinase (*Blk*) in iNKT17 cells which is involved in signalling via surface bound BCR on B cells (Fig. 3b). Although not being part of the TCR complex, BLK influences TCR signalling in γδT cells and it is crucially involved in development of γδT effector cells producing IL-17 (ref. 36). Thus, each subset of iNKT cells may have developed unique TCR-signalling features suitable to support their specialized effector functions and probably also their differentiation.

### iNKT17 cells can express IL-4 along with IL-17

**Figure 4:**
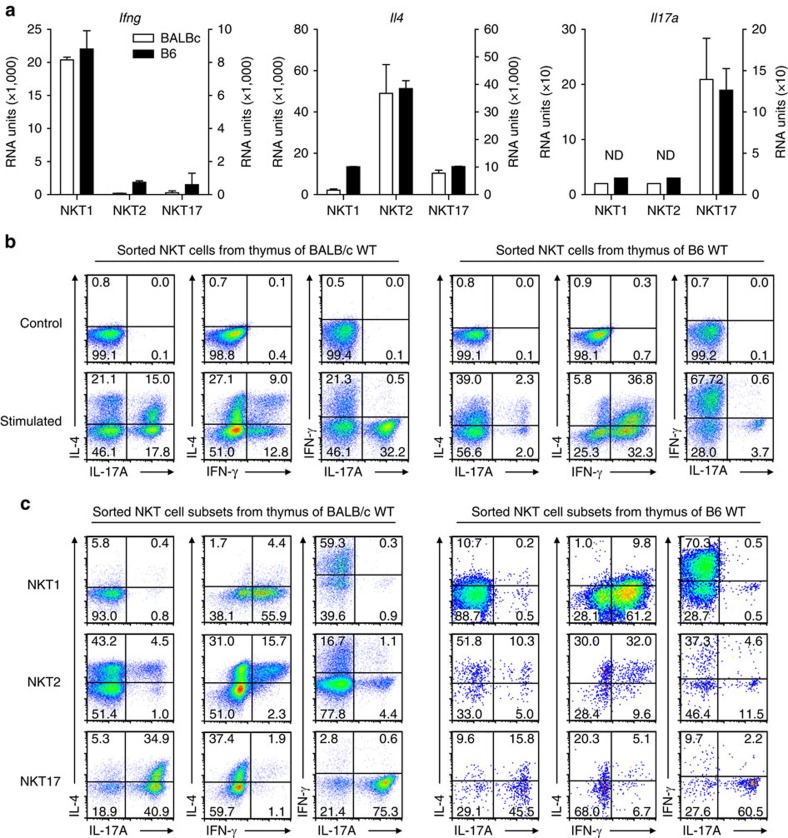
Expression of IL-4 and IFNγ and IL-17 in iNKT cells or their subsets. (**a**) mRNA expression profiles resulting from transcriptome analyses as depicted in Fig. 1e,f (mean±s.d., *n*=2). ND, none detected. (**b**) Total fractions of iNKT cells sorted from thymi of BALB/c or B6 mice, gated as shown in Fig. 1b. Sorted cells were kept either on ice (control) or stimulated with PMA/ionomycin in vitro in the presence of Brefeldin A (stimulated) before intracellular detection of the indicated cytokines by flow cytometry. (**c**) Detection of cytokines as described in **b** but iNKT cells from each individual thymus were sorted into iNKT1, 2, and 17 cells, respectively, by gating as shown in Fig. 1b. Pixel sizes in the plots to the right were increased to improve visibility. Data are representative of at least 4 (**b**) and 2 (**c**) independent experiments analyzing at least 4 (**b**) and 3 (**c**) individual thymi.

With respect to the key cytokines, IFNγ is strongly expressed in iNKT1 cells and IL-17 mRNA is only detectable in iNKT17 cells (Fig. 4a). Note that the signal intensity is low wherefore the bars visible for iNKT1 and iNKT2 cells represent background intensity and not bona fide signals. mRNA coding for IL-4 is not only present in iNKT2 cells but also in iNKT17 to a low yet significant extent rendering it unlikely that cross-contaminating iNKT2 were causing it. Since it is known that some iNKT cells express both, IL-4 and IFNγ, at the same time^37^,^38^, we applied a protocol allowing the simultaneous detection by flow cytometry of the three key cytokines IFNγ, IL-4 and IL-17 in PMA/ionomycin stimulated iNKT cells sorted from BALB/c and B6 thymi (Fig. 4b). The cytokine expression profiles of BALB/c iNKT cells differ profoundly from B6 cells but much of these differences are caused by the very different subset composition of the thymic iNKT cells (Supplementary Fig. 2b,c). For example, iNKT1 cells producing IFNγ prevail in B6 and therefore IFNγ^+^ cells are more frequent when compared with BALB/c (Fig. 4b). Cells of both strains have in common subpopulations expressing either cytokine alone as well as cells expressing IL-4 along with IFNγ but also cells expressing IL-4 and IL-17 simultaneously. Notably, cells expressing IFNγ and IL-17 are extremely rare in both, BALB/c and B6 thymus. To investigate more thoroughly the subtype origin of the cytokine single or double producing cells, iNKT cells sorted into iNKT1, 2 and 17 subpopulations (Fig. 1b) were stimulated with PMA/ionomycin and their cytokine expression profile assessed by flow cytometry (Fig. 4c). As expected, BALB/c iNKT1 cells produce IFNγ to a large extent and roughly 10% of these cells are IFNγ^+^IL-4^+^. Approximately 50% of BALB/c iNKT2 cells produce IL-4 and about 10% of these are IL-4^+^IL-17^lo-hi^. The IL-17 signal of these cells is not homogeneous but scatters along the IL-17 axis. A considerable percentage of iNKT2 cells are IFNγ^+^IL-4^+^. Approximately 70–80% of BALB/c iNKT17 cells are IL-17^+^ but roughly half of these cells also produce IL-4 although the signal intensity of the IL-4 signal among the double producers is lower than that observed in the iNKT2 IL-4^+^IL-17^lo-hi^ cells. The quite different appearance of the cytokine double producers, IL-4^+^IL-17^lo-hi^ versus IL-4^lo^IL-17^+^, present among iNKT2, and iNKT17 cells, respectively, makes it unlikely that they originated from mutual cross-contamination during iNKT sorts but rather identifies them as separate constituents of the iNKT2 and iNKT17 cell pool. This is supported by the observation that BALB/c iNKT2 cells expressing IL-17 are RORγt^−^ whereas all other IL-17^+^ iNKT cells (presumably iNKT17 cells) express RORγt (Supplementary Fig. 3a). The pronounced simultaneous production particularly of IL-4 and IL-17 by iNKT17 cells reported here may be provoked by the massive and somewhat artificial PMA/ionomycin stimulation. Therefore, sorted iNKT17 cells from BALB/c thymus were stimulated *in vitro* with anti CD3/CD28 antibody to mimic a more physiological setting. Expectedly, this results in a less robust cytokine production but also under these conditions, a population of IL-4^lo^IL-17^+^ cells is clearly detectable (Supplementary Fig. 3b). iNKT subpopulations sorted from B6 thymus show on PMA/ionomycin stimulation cytokine expression profiles that are similar to those obtained from the BALB/c cells although variations exist regarding the frequency of distinct cytokine expressing cells. However, the purity of sorted iNKT17 cells is lower than in case of BALB/c probably because the preponderance of iNKT1 cells present among B6 tet^+^ cells results in a certain degree of contamination during the sort. Neglecting these technical limitations, also in B6 it is evident that iNKT17 cells are composed of IL-17^+^ and IL-4^lo^Il-17^+^ cells. Moreover, iNKT2 cells encompass a small but significant fraction of IL-4^+^IL17^lo-hi^ cells.

### Expression of receptors required for migration

**Figure 5:**
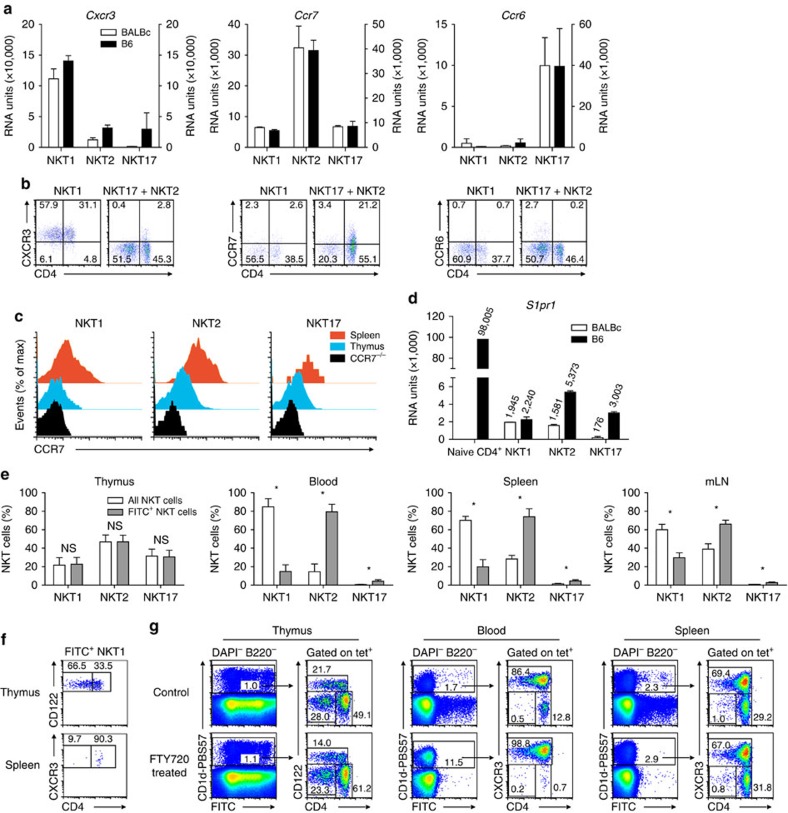
Expression of chemokine receptors and S1P1 by iNKT subsets. (**a**,**b**) Expression of chemokine receptors CXCR3, CCR7 and CCR6, (**a**) on the RNA level as depicted in Fig. 1e,f (mean±s.d., *n*=2) and (**b**) protein expression detected on the cell surface by antibody staining. Cells were gated as shown in Fig. 1b. (**c**) Expression of CCR7 by iNKT cells (DAPI^−^B220^−^tet^+^) of BALB/c thymus (blue) and spleen (red). Thymic iNKT cells of a CCR7^−/−^ animal served as a control. (**d**) mRNA levels coding S1P1 as depicted in Fig. 1e,f. Numbers given above bars represent average RU obtained by evaluation of transcriptome data. For a comparison, data from a different experiment analyzing naïve peripheral CD4^+^ cells of B6 are also shown (naïve CD4^+^). (**e**) BALB/c iNKT subtype composition including all iNKT cells (open bars) or only iNKT RTE (FITC^+^, grey bars) in compartments as indicated following a gating strategy as shown in Supplementary Fig. 2b. Shown are means±s.d. (*n*=4). (**f**) Expression of CD4 by BALB/c FITC^+^ iNKT1 cells (DAPI^−^B220^−^tet^+^) of thymus (upper panel) and spleen (RTE, lower panel). (**g**) BALB/c mice were gavaged with either PBS (control) or FTY720 and then FITC was injected into thymus one day later. Around 40 h. after injection the cells were analyzed in the indicated compartments. Gating was done as shown in Supplementary Fig. 2b. Data are representative of at least 3 (**b**), 3 (**c**), 2 (**e**), 2 (**f**) and 2 (**g**) independent experiments. An unpaired two-tailed Mann–Whitney test was performed in (**e**); NS, not significant (*P*>0.05), *P<0.05.

iNKT cells express various chemokine receptors and migrate in response to chemokines^18^. However, unlike naïve T cells iNKT cells are rather sessile and recirculate only modestly^17^. Therefore, for iNKT cells the chemokine/receptor-system may be of primordial importance for processes like intra-organ localization/retention or guiding iNKT cells freshly released from thymus to peripheral organs^39^,^40^. We find that all iNKT subsets express high levels of CXCR6 mRNA (Supplementary Fig. 2a)^18^. Expression of CXCR3 mRNA that is up-regulated by TBET^41^ is biased and high only in iNKT1 cells (Fig. 5a). Virtually all iNKT1 cells express the protein on their surface (Fig. 5b). This finding can be exploited to replace CD122 as a marker to identify iNKT subtypes since identical subset compositions regarding thymic iNKT cells of BALB/c or B6 origin were obtained when using either marker (Supplementary Fig. 2b,c). Also the purity of the CXCR3/CD4-gated subpopulations is comparable to that using CD122/CD4 (Supplementary Fig. 2d, compare with Fig. 1d). In periphery, CD122 expression on iNKT1 cells declines, whereas that of CXCR3 increases. Therefore, it is of advantage to use CXCR3 when classifying iNKT subtypes in periphery (Supplementary Fig. 2e). CCR7 is detectable only on the surface of approximately one-third of iNKT2 cells and only very few iNKT1 and iNKT17 cells are CCR7^+^ (Fig. 5a,b). In addition, CD62L is expressed only marginally on iNKT2 cells^18^ that should render homing via HEV exploiting the classical CCR7/CD62L-based system inefficient. Instead, peripheral iNKT cells increase expression of CCR7 (Fig. 5c), whereas that of CD62L remains low (Supplementary Fig. 2f). However, iNKT cells express significant levels of mRNA coding P-selectin ligand (*Selplg*/PSGL-1, Supplementary Fig. 2g). By binding to CCL19 and CCL21 PSGL-1 reinforces CCR7 mediated homing of T cells to lymph nodes via HEV obviating the need of CD62L for this process^42^. Thus, PSGL-1 could confer both, iNKT migration into lymph nodes as well as to peripheral tissue. iNKT17 cells express specifically CCR6 mRNA as reported earlier^40^, but on only a small subpopulation of iNKT17 cells from BALB/c thymus CCR6 is detectable on the cell surface (Fig. 5a,b).

It was shown that iNKT cells that lack the sphingosine-1-phosphate receptor 1 (S1P1) develop normally in thymus but iNKT cell numbers are severely reduced in periphery^43^. Therefore, S1P1 expression governs exit of iNKT cells from thymus as in case of mature naive thymocytes but the block of emigration is not complete. Surprisingly, the level of S1P1 mRNA expression in iNKT cells is low particularly when compared with that of naive CD4^+^ T cells (Fig. 5d). In BALB/c but not B6 iNKT17 cells the signal reflecting S1P1 mRNA expression is coming close to background levels. This may render emigration of iNKT17 inefficient providing a potential explanation why iNKT17 cells are more frequent in BALB/c thymus as compared with B6 (Supplementary Fig. 2b,c). To test whether low S1P1 mRNA levels still efficiently control exit, we first analyzed recent thymic emigrants (RTE). Indeed, iNKT17 cells are very rare among iNKT RTE although more frequent in B6 than in BALB/c (Fig. 5e; Supplementary Fig. 4 for B6). However, there is no simple linear correlation of S1P1 mRNA levels with emigration rate also because irrespective of the mRNA level, iNKT2 cells emigrate at considerably higher rates than the other subsets. This is in agreement with earlier observations demonstrating that immature iNKT cells preferentially exit thymus. Unexpectedly however, most of the emigrating iNKT1 cells are CD4^+^ (Fig. 5f). Treatment of BALB/c mice with FTY720, a drug that mimics absence of S1P1, before FITC injection is done, causes a >95% drop in frequency of CD4^+^ RTE among splenic lymphocytes demonstrating the effectivity of drug application (Supplementary Fig. 5a). At the same time, only very few FITC^+^ iNKT cells are present in periphery (Fig. 5g; Supplementary Fig. 5a) confirming that S1P1 expression by all iNKT cells is mandatory for thymus exit. Surprisingly, FTY720 treatment causes an almost complete disappearance of iNKT2 but not iNKT1 cells from circulation (Fig. 5g, blood versus spleen, summary in Supplementary Fig. 5b) indicating a very short dwell time of iNKT2 cells in blood.

### NK-characteristic genes are expressed by iNKT1 cells

**Figure 6:**
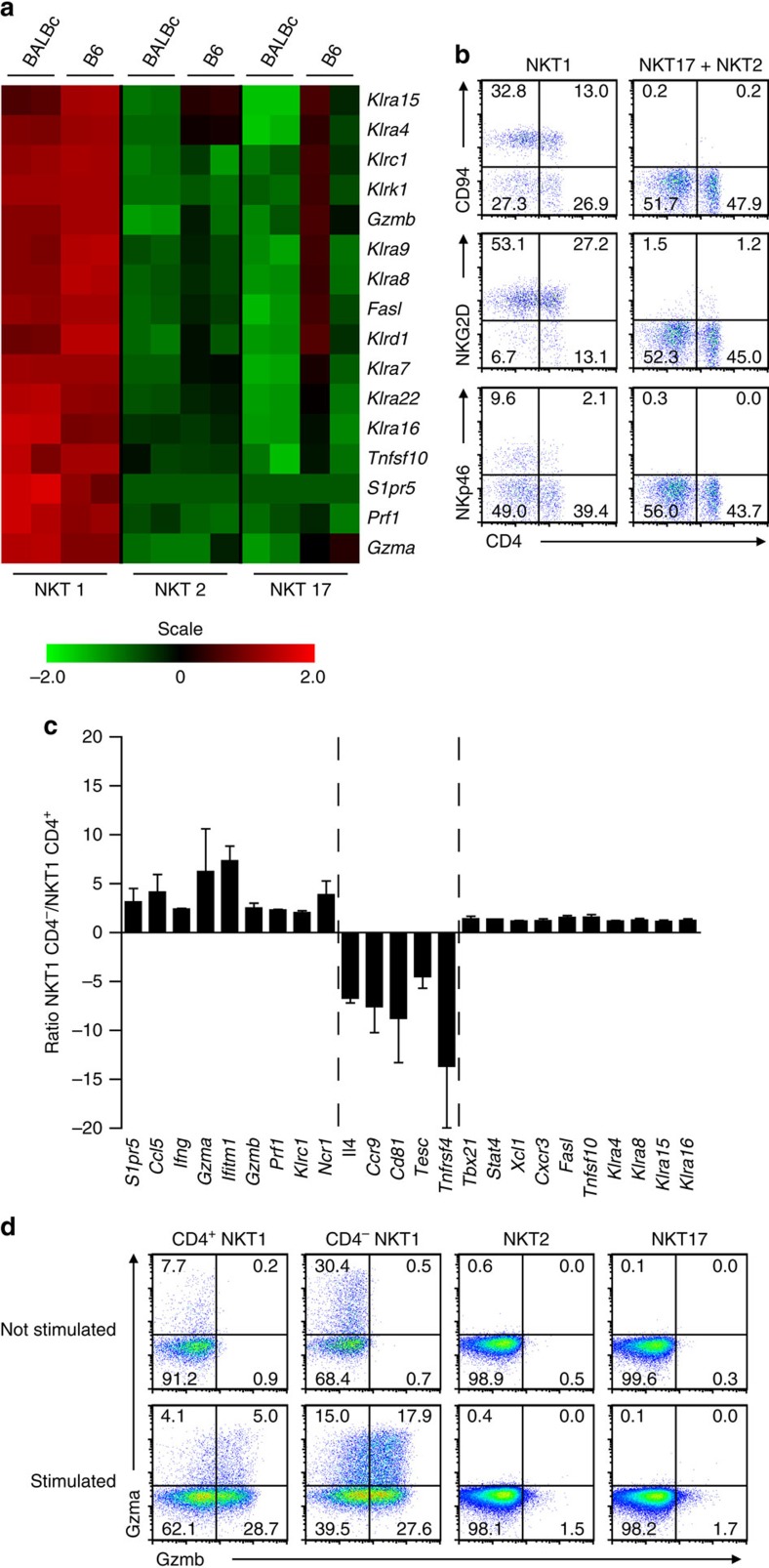
CD4^+^iNKT1 and CD4^−^iNKT cells differ in their NK-like phenotype. (**a**) Heat map of selected genes with known relevance for NK cell function. Gene names are given on the right side. Data source as in Fig. 1e,f (*n*=2). (**b**) Cell surface expression of CD94 (*Klrc1*), NKG2D (*Klrk1*) and NKp46 (*Ncr1*) in iNKT subsets of BALB/c thymus, gating was done as shown in Fig. 1b. (**c**) Expression of selected genes in iNKT1 subpopulations that were sorted in two fractions according to their CD4 expression. Data were obtained following two independent transcriptome analyses. Depicted are the mean ratios (±s.d.) of mRNA levels as indicated (*n*=2). (**d**) iNKT cells of BALB/c thymus were sorted into subpopulations as indicated (top row) according to the gating strategy shown in Fig. 1b. Cells from 3 thymi were pooled for each sort and sorted cells either kept on ice (not stimulated) or stimulated with PMA/ionomycin for 4 h. before intracellular detection of granzyme a and granzyme b. Data are representative of 3 (**b**) and 2 (**d**) independent experiments.

Compared with NK cells there is only a very limited number of reports investigating the (NK-like) killing capacity of iNKT cells (for example, refs 44, 45, 46). However, *in vivo* it is difficult to study direct killing mediated by iNKT cells because of their capacity to propel the killing activity of NK cells^47^,^48^ that was shown to be mediated by IFNγ^49^ or IL-2 (ref. 48) produced by activated iNKT cells. We find that the bulk of NK signature genes are expressed by iNKT1 cells (Fig. 6a) confirming the tendency of increased transcription of these genes when iNKT cell mature into NK1.1^+^ cells^24^. Indeed, the segregation of NK-specific transcripts/proteins with iNKT1 but not the other two subsets is remarkably complete particularly in case of BALB/c (Fig. 6a,b,d; Supplementary Fig. 6) lending impressive support to the hypothesis that discrimination of iNKT cells into iNKT1, 2 and 17 cells reflects functional differences between these subsets. However, iNKT1 cells may not represent a uniform population as reflected already by their broadly diverging expression of CD4. Therefore, we asked whether a disequilibrium exists in expression between CD4^+^iNKT1 and CD4^−^iNKT1 cells especially with respect to NK-relevant genes. We sorted BALB/c iNKT1 cells into CD4^lo-int^ and CD4^int-hi^ cells and performed chip hybridization. Two independent experiments were done (GEO accession number 69120). NK-marker genes are either expressed much stronger in CD4^−^iNKT1 cells or equally well in CD4^+^iNKT1 and CD4^−^iNKT1 cells (Fig. 6b,c). This latter group not only contains genes like *Tbx21*, *Stat4* or *Xcl1* but also encompasses many NK-receptors (Fig. 6c). NK-marker genes with preferential expression in CD4^+^iNKT1 cells are not detectable. These cells preferentially express genes unrelated so far to NK-function or even counter-acting it such as CD81 (ref. 50) or IL-4 (ref. 51). *Gzma* (granzyme a) and *Gzmb* (granzyme b) represent key genes involved in NK-triggered killing, and we observe their expression primarily in CD4^−^iNKT1 cells. When investigating their expression on the protein level in more detail on iNKT cells sorted into CD4^−^iNKT1, CD4^+^iNKT1, iNKT2 and iNKT17 cells, respectively, granzyme a is already detectable in steady state in iNKT1 cells whereas granzyme b is translated into protein only on stimulation. In both cases, CD4^−^iNKT1 cells produce significantly more granzymes than the CD4^+^ cells. Taken together, these results suggest that a NK-like phenotype is not restricted to the CD4^−^iNKT1 cells but their more pronounced equipment with NK-specific machinery may accentuate the NK-character of CD4^−^iNKT1 cells.

### SerpinB1 impacts on iNKT17 cell numbers in thymus and lung

**Figure 7:**
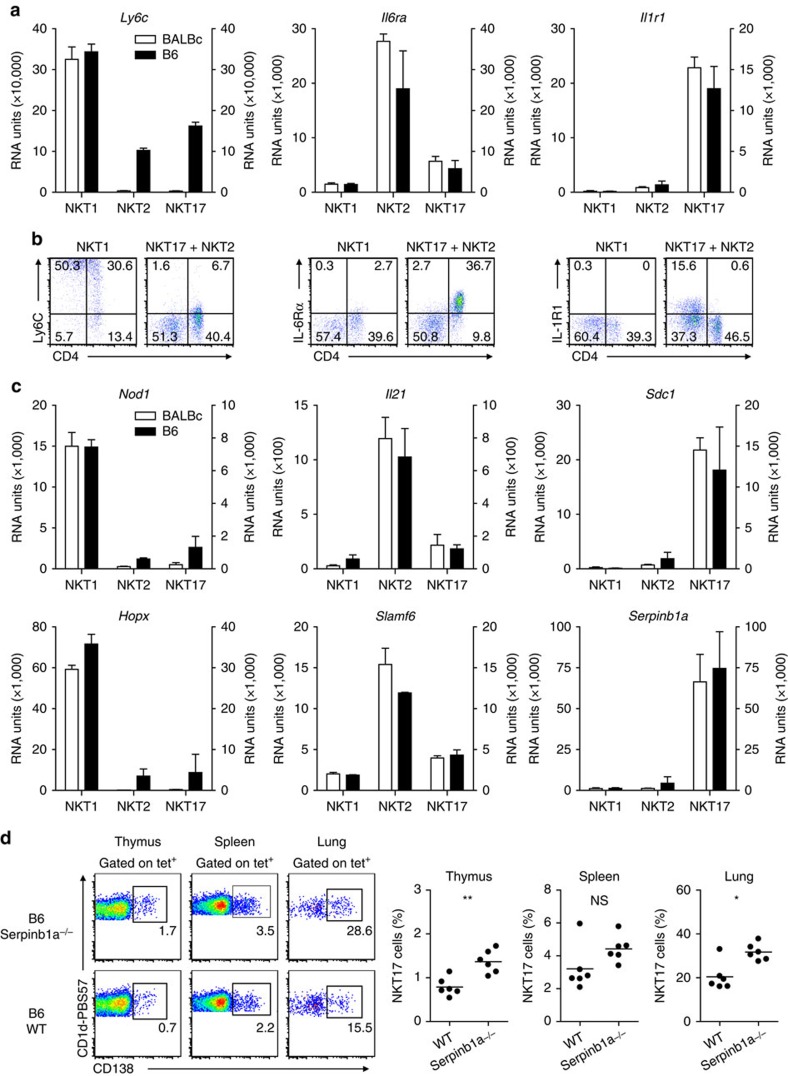
SerpinB1 deficiency impacts on frequency of iNKT17 cells. (**a**,**c**) mRNA levels coding for selected genes that are expressed in a iNKT subtype specific fashion as depicted in Fig. 1e,f (mean±s.d., *n*=2). (**b**) Cell surface expression of Ly6c (left panel), IL-6Rα (middle panel) and IL-1R1 (right panel) on iNKT1, 2 and 17 cells (DAPI^−^B220^−^tet^+^) isolated from thymus of BALB/c mice. (**d**) iNKT17 cells are expanded in frequency in various organs of *Serpinb1a*^−*/*−^ mice. Left panels show a representative stain of iNKT17 cells (DAPI^−^B220^−^tet^+^) of various organs of *Serpinb1a*^−*/*−^ or WT mice based on the expression of syndecan-1 (CD138). Right panels: Each dot represents one animal analyzed (WT: *n*=6; ko: *n*=6). Unpaired two-tailed *t*-test was done for thymus, two-tailed Mann–Whitney test for spleen and lung. NS, not significant (*P*>0.05), **P<0.01. Data are representative of 2 (**b**,**d**) independent experiments or were pooled from 2 independent experiments (**d**, right panels).

Our initial studies exploring iNKT cells from BALB/c thymus revealed many additional genes that exert a biased expression profile among the iNKT subsets (Supplementary Table 2). Figure 7 shows a compendium of further genes that display a subtype-specific expression pattern. The functional relevance of these genes for iNKT cell biology was partly investigated^52^,^53^,^54^,^55^,^56^ but the chip analyses performed also reveal some interesting candidates for further research. *Hopx* (Fig. 7c) codes for a transcription cofactor that affects function of regulatory T cells^57^ and influences survival of TH1 cells^58^. The serine protease SerpinB1 regulates expansion of IL-17 producing γδT cells and Th17 cells^59^. Its strong expression by iNKT17 cells led us to speculate whether SerpinB1 controls abundance of these cells. We detected iNKT17 cells from organs of serpinb1a^−/−^ mice based on their specific expression of syndecan-1 (Fig. 7c,d)^52^. While total frequencies of iNKT cells in thymus, spleen and lung are not affected in serpinb1a^−/−^ animals, those of iNKT17 are increased in all organs tested.

## Discussion

Recent studies suggest that the subdivision of iNKT cells into iNKT1, 2 and 17 cells represents a well-founded platform to explain many of the variegated immunological activities of these cells. We investigated this in more detail performing a series of transcriptome analyses of sorted iNKT subpopulations. We identify many genes expressed highly specific in either of the BALB/c iNKT1, 2 or 17 subsets. This is not only obvious for NK-related markers expressed by iNKT1 cells but also other genes of known or hitherto unknown relevance for iNKT cell development and function. However, the frequently observed unambiguous correlation of gene expression with iNTK subtype is on average less pronounced in B6. We assume that this is caused by the less clean cell sorts because the predominant iNKT1 cell population complicates proper gating. Advanced sorting strategies making use of alternative markers like CXCR3 or syndecan-1 will be useful to bypass this problem in future analyses. Despite of this, the expression pattern of numerous iNKT-key genes is highly similar between both mouse strains.

The frequency of iNKT cells as well as the composition of the iNKT cell pool in thymus varies greatly depending on the mouse strain under scrutiny^23^,^60^. iNKT cells are less frequent in B6 thymus with iNKT1 cells predominating whereas in BALB/c iNKT2 cells prevail but also prominent subpopulations of iNKT1 and iNKT17 cells are present. The genetic background is likely to play a major role governing the subset composition even though exogenous factors were also described to contribute^61^. We found several genes that are expressed quite differently in B6 versus BALB/c iNKT cells that may help explain the existing variations among mouse strains. For example, mRNA for cathepsin L is strongly expressed in BALB/c but not B6 iNKT cells (Fig. 2). Mice deficient for cathepsin L almost completely lack iNKT cells^62^ and the divergent levels observed in iNKT cells hint to a potential role of cathepsin L in governing the quantity of iNKT cells generated in thymus of different mouse strains. A strain specific expression is also evident for *Hey1* coding for HESR1, a transcriptional repressor, and *Ascl1* (MASH1), a transcriptional activator. In neuronal cells, expression of HESR1 correlates with delayed differentiation of progenitors and functionally antagonizes MASH1 that promotes their differentiation^63^. It is possible, that this transcriptional network is also involved in regulating the dynamics of iNKT cell differentiation making them promising candidates for future work exploring iNKT cell development and function.

Expression of functional S1P1 is necessary but not sufficient for iNKT cells to leave thymus. Among RTE, iNKT2 cells are present at a disproportionally high frequency although iNKT1 and iNKT2 cells express similar levels of S1P1 mRNA. Although additional factors may render iNKT2 cells more competent to emigrate, it is likely that iNKT1 cells are hampered in emigration by high levels of CXCR3 expression as already described earlier^64^,^65^. Nevertheless, iNKT1 cells are present among RTE to a considerable extent. Remarkably however, these iNKT1 cells were mostly CD4^+^ indicating that CD4^−^ cells don't emigrate efficiently. The cause for this remains an open issue since both subpopulations express comparable amounts of mRNAs coding for S1P1 and CXCR3. It was demonstrated that iNKT RTE in juvenile mice rather quickly develop from NK1.1^−^ into NK1.1^+^ cells^15^ and that CD4^+^IL-4^−^iNKT2 cells can differentiate into CD4^+^iNKT1 cells^23^. Therefore, a distinct proportion of the iNKT1 RTE may represent cells that emigrate as iNKT2 cells but convert into iNKT1 cells in periphery (maintaining their CD4 expression). However, this cannot explain the rapid and almost complete disappearance of iNKT2 cells from blood following FTY720 administration since the proportion of iNKT2 cells in spleen remains unchanged. The almost complete clearance from blood rather indicates that iNKT2 cells can recirculate via CCR7/PSGL-1 and are trapped inside lymph nodes on FTY720 treatment just as naïve T cells. iNKT cells are rather sessile and hardly recirculate^17^,^66^ but these analyses investigated the total pool peripheral iNKT cells where iNKT2 cells are much less frequent than iNKT1 cells. Interestingly, the ‘highest' exchange rate for iNKT cells in parabiotic mice occurs in peripheral lymph node where iNKT2 tend to migrate preferentially supporting the idea that iNKT2 cells but no other subsets possess a limited recirculation potential^17^,^60^.

Cytotoxic cells can execute killing of targets by either membrane bound receptor systems such as FasL/Fas or TRAIL/TRAILR or via soluble components like perforin, granzyme a and granzyme b. Soluble mediators are expressed preferentially by CD4^−^iNKT1 cells whereas some NK-receptors as well as Fas ligand (*fasl*) and TRAIL (*Tnfsf10*) display a much more balanced expression not co-varying with CD4-levels. Therefore, CD4^+^iNKT1 cells should be able to kill target cells. This is supported by a recent report suggesting that antigen dependent killing by iNKT cells is mediated via the FASL/FAS-axis^46^ and correlates positively with TCR-signalling strength. If so, this would handicap the killing capacity of CD4^−^iNKT1 cells because they profit much less from benefits of CD4-mediated amplification of the TCR signal than the CD4^+^ cells^29^. On the other hand, it was shown that CD4^−^iNKT cells isolated from liver are far more potent in tumour rejection than CD4^+^iNKT cells^51^ and also express significantly more IFNγ and granzymes (this report). Interestingly, the granzymes possess additional functions: Granzyme a can promote inflammation by inducing secretion of IL-1β, TNFα and IL-6 by cells exposed to it^67^ and granzyme b plays a role in remodelling basement membranes of endothelia supporting efficient transmigration of effector cells to sites of inflammation^68^. Taken together, this suggests that the two iNKT1 subpopulations perform overlapping but also specialized jobs in immune surveillance.

Surprisingly, we observed that in BALB/c but also in B6 up to half of the thymic iNKT17 cells express low levels of IL-4 along with IL-17. This population escaped detection in earlier studies probably because iNKT17 cells are much less frequent in B6 thymus. In an attempt to explore differences between IL-17^+^iNKT17 and IL-17^+^IL-4^+^iNKT17 cells, we failed to detect a surface receptor that would allow cell sorting of these two populations. Therefore, we investigated 4get mice where IL-4 expression is coupled to that of GFP due to a knock-in into the genomic IL-4 locus. However, the GFP-signal detected in thymic iNKT17 cells of these mice is smeary and cells lacking any GFP-signal represent a small minority (Supplementary Fig. 2h). This is consistent with the assumption that IL-17^+^IL-4^+^iNKT17 cells represent an intermediate commencing final differentiation into single positive IL-17^+^iNKT17 cells. This idea suggests further that developmental precursors of IL-17^+^IL-4^+^iNKT17 cells originate from the pool of iNKT2 cells. In support of this, we detect a small but distinct population of cells among iNKT2 cells that is characterized by an IL-4^+^IL-17^lo-hi^ signal. Along with recent findings^23^,^27^ this places iNKT2 cells in the centre of final differentiation of iNKT17 and iNKT1 cells. In line with this, prominent fractions of IL-4^+^IFNγ^+^ and IL-4^+^IL-17^+^ cells are only present in the iNKT2 cell pool and at the same time a substantial population of IL-17^+^IFNγ^+^ cell was never detected.

Although the results shown here provide comprehensive support for the iNKT1/2/17-concept, the in depth characterization of iNKT1, 2 and 17 thymocytes reveals that each subset is not composed of a uniform population of cells. This was already shown to be the case for iNKT2 cells where IL-4^+^ cells are clearly distinct from IL-4^−^ cells. We provided evidence that a substantial proportion of iNKT17 cells express IL-4 along with IL-17. It remains to be determined whether IL-4^+^IL-17^+^ iNKT cells represent a transitory population differentiating into IL-17^+^ cells. Our current view would be in line with a hypothesis merging traditional and new aspects as already proposed before^23^,^27^,^69^: each iNKT subset contains a population of terminally matured cells (CD4^−^iNKT1, IL-4^+^iNKT2, IL-17^+^ iNKT17) but also a population of cells not yet finally differentiated (CD4^+^iNKT1 cells, IL-4^−^iNKT2, IL-4^+^IL-17^+^iNKT17).

## Methods

### Mice

C57BL/6N (B6) and BALB/cAnNCrl (BALB/c) mice were bred in the animal facility of Hannover Medical School under specific pathogen-free conditions. B6.Serpinb1a^tm1.1Cben^ mice referred to as serpinB1^−/−^ mice throughout the manuscript, and the corresponding control mice were raised at the University of Bern. BALB/c.129-Il4^tm1Lky^ mice referred to as 4get mice were raised at the University of Erlangen, C.129P2(B6)-Fcer1g^tm1Rav^ (FceR1g^−/−^) were purchased from Taconic Biosciences and the respective control BALB/cByJ animals from Charles River Laboratories. All animals used in this study were female and were 7–12 weeks old at the time of analysis. All animal experiments were approved by the LAVES, Lower Saxony, and were conducted according to MHH guidelines.

### Lymphocyte isolation from lung and liver

Lungs and Livers were perfused with cold PBS before harvest. Organs were cut into small pieces and digested with 0.5 mg ml^−1^ collagenase D (Roche) and 0.025 mg ml^−1^ DNase I (Roche) in RPMI 1640/5% FCS for 45 min at 37 °C. After digestion EDTA was added to a final concentration of 20 mM. Next, samples were mashed trough 40 μm cell strainers and washed once in RPMI 1640 medium/5% FCS. Samples were layered on Percoll gradients (40–70%) and centrifuged for 20 min at 300*g*. Following centrifugation, the interphase containing the enriched lymphocytes was collected.

### Flow cytometry

Single-cell suspensions were prepared and blocked with 3% rat serum in FACS buffer (PBS/3% FCS). All surface stainings were performed for 30 min on ice, except for CCR7 which was done for 30 min at 37 °C. Intracellular stainings for PLZF and RORγt were done using Foxp3 staining buffer (eBioscience), according to the protocol provided by the manufacturer. For intracellular cytokine stainings the ICS staining buffer set (eBioscience) was used. Antibodies used (clone name; dilution): anti-mCD4 (RM4–5; 1:400), anti-mCD122 (TM-β1; 1:200), anti-mLy6C (HK1.4; 1:400), anti-mIL4 (11B11; 1:100), anti-mIL6Rα (D7715A7; 1:100), anti-mIL1R (JAMA-147; 1:100), anti-mCCR6 (29–2L17; 1:100), anti-mNKG2D (CX5; 1:200), anti-mIFNγ (XMG1.2; 1:100) and anti-m IL17A (TC11-18H10.1; 1:1,000) all from BioLegend; anti-mCD3ɛ (145-2C11; 1:100) anti-mPLZF (Mags21F7; 1:100), anti-mRORγτ (B2D; 1:400), anti-mCXCR3 (CXCR3-173; 1:100), anti-mCCR7 (4B12; 1:100), anti-mGzma (GzA-3G8.5; 1:100), anti-mGzmb (NGZB; 1:100) and anti-mB220 (RA3-6B2; 1:200) were from eBioscience. Anti-mCD94 (18d3; 1:100) and anti-mNKp46 (29A1.4.9; 1:100) were from Miltenyi Biotec, anti-mCD247 (H146-968; 1:100) was from Molecular Probes and anti-mCD138 (281-2; 1:200) was from BD Pharmingen. CD1d tertramer loaded with PBS57 (analogue of α-galactosylceramide) was provided by the tetramer facility of US National Institutes of Health and used in a dilution of 1:1000. Data were acquired on LSR II (Becton and Dickinson) and analyzed using FlowJo (TreeStar).

### *In vitro* cytokine production

Sorted thymic iNTK cells were plated at a density of 5 × 10^5^ per ml and incubated at 37 °C for 4 h in presence of ionomycin (1.5 μg ml^−1^), PMA (phorbol 12-myristate 13-acetate; 50 ng ml^−1^) and Brefeldin A (10 μg ml^−1^) in RPMI 1640/10% FCS. During this step control cells are kept on ice in FACS buffer. For some experiments, 200 μl of sorted iNKT17 cells in RPMI 1640/10% FCS/10 μg ml^−1^ Brefeldin A were seeded into wells that were coated with 1 μg ml^−1^ anti CD3 antibody (clone 17A2) and 2 μg ml^−1^ anti CD28 antibody (clone 37.51). Cells were incubated for 6 h at 37 °C before cytokine detection by intracellular staining.

### Intrathymic FITC injection

Before injection, mice were anaesthetised by an intra-peritoneal injection of xylazine (9 mg per kg body weight) and ketamine (90 mg per kg body weight). Each thymic lobe was injected with 10 μl 500 ng ml^−1^ FITC solution in PBS (Sigma-Aldrich) using a 27 G needle. Forty hour post-injection cells from blood, spleen and mLN were analyzed.

### Cell sorting

Single-cell suspensions from single or pooled thymi were prepared, stained with appropriate Ab and sorted on FACSAria IIu or FACSAria Fusion (Becton Dickinson). Cell fractions were collected as follows: iNKT (DAPI^−^B220^−^CD1d-PBS57^+^), iNKT1 (DAPI^−^B220^−^PBS57^+^CD122^+^), iNKT2 (DAPI^−^B220^−^CD1d-PBS57^+^CD122^−^CD4^+^) and iNKT17 (DAPI^−^B220^−^CD1d-PBS57^+^CD122^−^CD4^−^). The purity of sorted cells was routinely >90%.

### Isolation of RNA and microarray assay

Tymic iNKT subsets from pooled 5–6 female WT animals were FACS sorted and RNA was isolated using the RNeasy Plus Micro Kit (Qiagen).

The Microarray study has been performed by use of a refined version of the Whole Mouse Genome Oligo Microarray 4 × 44 K v2 (Design ID 026655, Agilent Technologies), called ‘026655AsQuadruplicatesOn4x180K' (Design ID 048306) developed in the Research Core Unit Transcriptomics of Hannover Medical School. Microarrays of this design type cover roughly 32000 murine transcripts. Microarray design was defined at Agilent's eArray portal using a 4 × 180 K design format for mRNA expression as template. All non-control probes of design ID 026655 have been selected to be printed four times onto one 180 K Microarray (yielding on-chip quadruplicate Features). Control probes required for proper Feature Extraction software operation were determined and placed automatically by eArray using recommended default settings.

A total of 6.5 ng RNA were used to prepare aminoallyl-UTP-modified (aaUTP) cRNA (Amino Allyl MessageAmp II Kit; #AM1753; LifeTechnologies) as directed by the company (applying one-round of amplification). The labelling of aaUTP-cRNA was performed by use of Alexa Fluor 555 Reactive Dye (#A32756; LifeTechnologies). Before the reverse transcription reaction, 1 μl of a 1:100,000 dilution of Agilent's ‘One-Colour spike-in Kit stock solution' (#5188–5282, Agilent Technologies) was added to each total RNA sample.

cRNA fragmentation, hybridization and washing steps were carried-out as recommended in the ‘One-Colour Microarray-Based Gene Expression Analysis Protocol V5.7', except that 300 ng (Exp1) or 130 ng (Exp2) of each fluorescently labelled cRNA population were used for hybridization.

Slides were scanned on the Agilent MicroArray Scanner G2565CA (pixel resolution 3 μm, bit depth 20). Data extraction was performed with the ‘Feature Extraction Software V10.7.3.1' using the extraction protocol file ‘GE1_107_Sep09.xml'.

Processed intensity values of the green channel, ‘gProcessedSignal' (gPS) were normalized by global linear scaling: All gPS values of one sample were multiplied by an array-specific scaling factor. This factor was calculated by dividing a ‘reference 75th Percentile value' (set as 1500 for the whole series) by the 75th Percentile value of the particular Microarray to be normalized (‘Array I' in the formula shown below). Accordingly, normalized gPS values for all samples (microarray data sets) were calculated by the following formula:


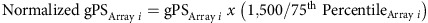


Measurements of on-chip replicates (quadruplicates) were averaged using the geometric mean of normalized gPS values to retrieve one resulting value per probe and sample. Single Features were excluded from averaging, if they (i) were manually flagged, (ii) were identified as Outliers by the Feature Extraction Software, (iii) lie outside the interval of ‘1.42 × interquartile range‘ regarding the normalized gPS distribution of the respective on-chip replicate population, or, iv) showed a coefficient of variation of pixel intensities per Feature that exceeded 0.5. A lower intensity threshold (surrogate value) was defined based on intensity distribution of negative control features. This value was fixed to 20. All of those normalized gPS values that fell below this intensity border were substituted by the respective surrogate value of 20.

### Bioinformatics and statistical analysis

The normalized gPS values for selected genes were used to generate heat maps using Qlucore Omics Explorer software tool. Prism (GraphPad) software was used for all statistical analysis performed in the current study. Statistical methods have not been used to predetermine sample sizes. Where appropriate, data were displayed as mean±s.d. as indicated in the legends to the figures, *n* denotes the number of data points. For all statistical evaluations a normality test and a variance test were done. Data sets displaying results from chip hybridizations were excluded from statistical comparisons because results are of inferior validity when comparisons of two versus two data points are done. For the statistical evaluations shown in Figs 3c, 5e and 7d and Supplementary Figs 1b,d,2c,4 and 5b three to eight animals per group as given in the legends were analyzed while allele status was known during analysis. Data were collected from at least two independent experiments and data obtained from all animals analyzed are shown. A two-tailed Mann–Whitney *U* test was done when *n* was ≤4 in one or both of the groups to be compared. For *n*>4 a Kolmogorov–Smirnov test was done checking for normal or non-normal distribution of the data points in each group. According to the results of the normality test either unpaired two-tailed *t*-test (normal distribution) or two-tailed Mann–Whitney *U* test (non-normal distribution) was used as indicated in the legends to the figures.

### Data availability

Transcriptome data that support the findings of this study have been deposited in GEO with the primary accession code GSE69120. The authors declare that all other data supporting the findings of this study are available within the article and its Supplementary Information Files.

## Additional information

**How to cite this article:** Georgiev, H. *et al*. Distinct gene expression patterns correlate with developmental and functional traits of iNKT subsets. *Nat. Commun.* **7,** 13116 doi: 10.1038/ncomms13116 (2016).

## Supplementary Material

Supplementary Information Supplementary Figures 1-6 and Supplementary Tables 1-2
