# Supplementary material for: Distinct gene expression patterns correlate with developmental and functional traits of iNKT subsets
Source: Nat Commun. 2016 Oct 10;7:13116. doi: 10.1038/ncomms13116 (PMC5062562; doi:10.1038/ncomms13116)
Supplement: Supplementary Information — Supplementary Figures 1-6 and Supplementary Tables 1-2 [file ncomms13116-s1.pdf]

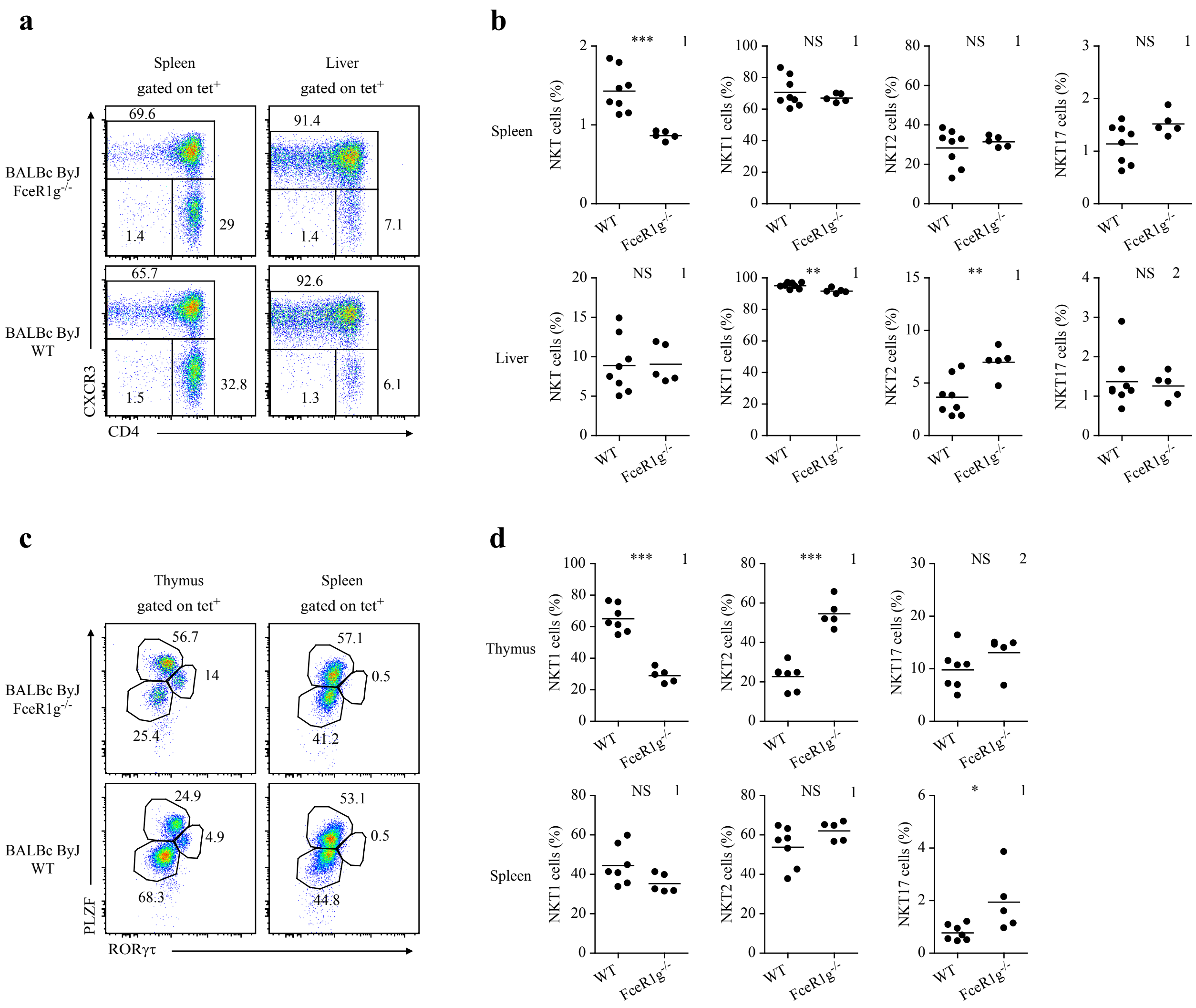

Supplementary Figure 1. Analysis of Fcer1g-/- mice.

(a) iNKT cells (DAPI-B220-tet+) isolated from spleen and liver of WT and Fcer1g-/- mice were gated according to their expression of CXCR3 and CD4. Numbers given indicate the percentage of cells in each gate. **(b)** Summary of data obtained from iNKT subset analyses as indicated in (a). **(c)** iNKT cells (DAPI-B220-tet+) from thymus and spleen were analyzed based on their expression of PLZF and RORγt. **(d)** Summary of data obtained from iNKT subset analyses as indicated in (c). The data shown in **(a, c)** are representative of at least 5 individually stained samples each, spleen and liver or thymus and spleen, respectively. Data shown in the right panels of **(b, d)** were collected from 2 independent experiments (WT: n=8; ko: n=5). Each dot represents one animal. The statistical tests performed are indicated by a small number in the upper right corner. 1: Unpaired two-tailed t-test, 2: Unpaired two-tailed Mann-Whitney test. NS, not significant ($P > 0.05$), *$P < 0.05$, **$P < 0.01$ and ***$P < 0.001$.

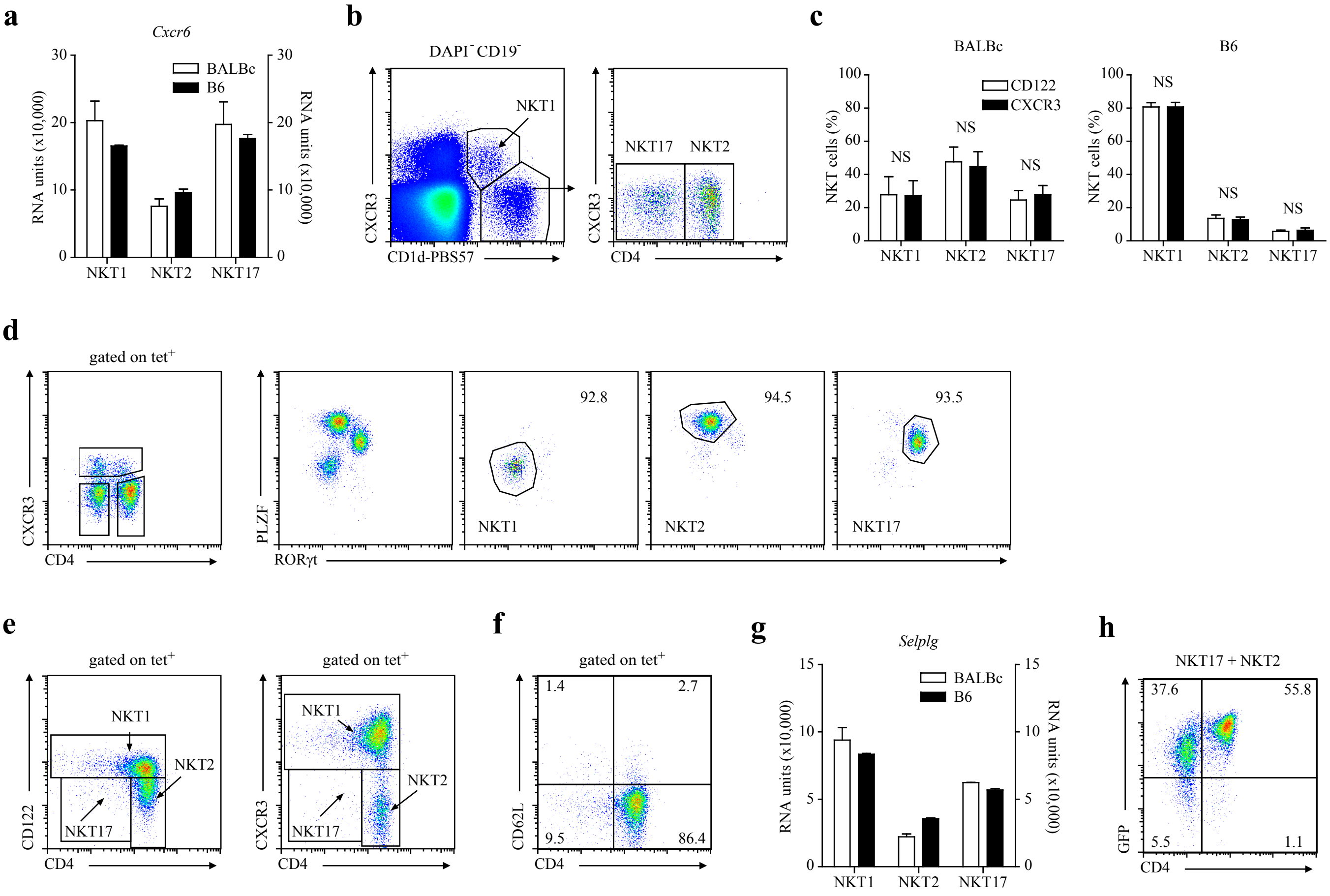

Supplementary Figure 2. CXCR3 as a marker to define iNKT1 cells

(a, g) Expression of the indicated genes in iNKT subsets depicted as in Fig. 1e, f. **(b)** Gating strategy for DAPI$^-$CD19$^-$ thymcytes as depicted in Fig. 1b but replacing CD122 by CXCR3. **(c)** Antibody staining of thymocytes using either expression of CXCR3 (black bar) or CD122 (open bar) for determining iNKT subset composition as shown in (b). Cell suspensions were analyzed in split samples of each thymus using either anti CXCR3 antibody or anti CD122 antibody. Shown are means ± SD (n=6-7) **(d)** iNKT1, 2 and 17 populations were gated according to expression of CD4 and CXCR3 and their purity determined by staining for PLZF and RORγt. **(e)** tet$^+$CD19$^-$DAPI$^-$ iNKT cells of BALB/c spleen can be subtyped into iNKT1, 2 and 17 cells more conveniently when using CXCR3 as marker (right panel) instead of CD122 (left panel). **(f)** Expression of CD62L on splenic iNKT17 and iNKT2 cells (tet$^+$DAPI$^-$B220$^-$CD122^{lo}). **(h)** Expression of GFP in iNKT17 and iNKT2 cells of thymus of 4get mice. Cell gating as in (f). Data are representative of at least 3 **(b, c, d, e)** independent experiments. An unpaired two-tailed t-test was performed in (c). NS, not significant (*P* > 0.05).

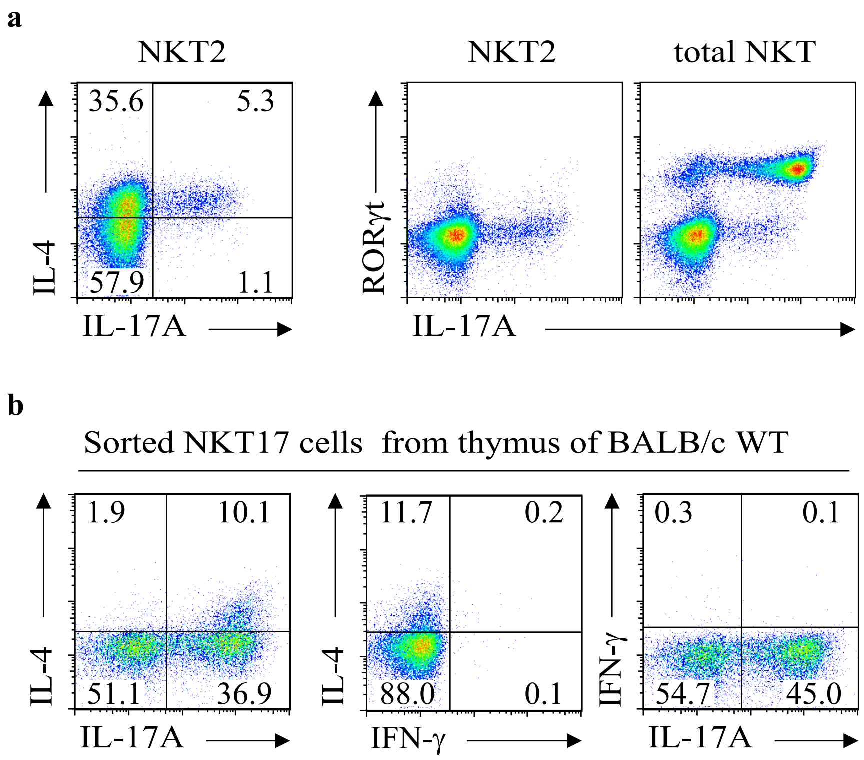

Supplementary Figure 3. iNKT subtype specific expression characteristics.
(a) All iNKT cells or iNKT2 cells were sorted from thymus of BALB/c mice and stimulated with PMA/ionomycin followed by simultaneous intracellular detection of IL-4 and IL-17 (iNKT2 cells, left panel) as described in Methods. In parallel samples, expression of IL-17 and RORγt was monitored in either iNKT2 cells or the pool of total iNKT cells (two panels to the right). **(b)** iNKT17 cells were sorted from BALB/c thymus and seeded onto 96well plates coated with anti CD3 and anti CD28 antibody (Methods). Production of cytokines was recorded by intracellular staining 6h later. Data shown are representative for at least two independently sorted cell populations each.

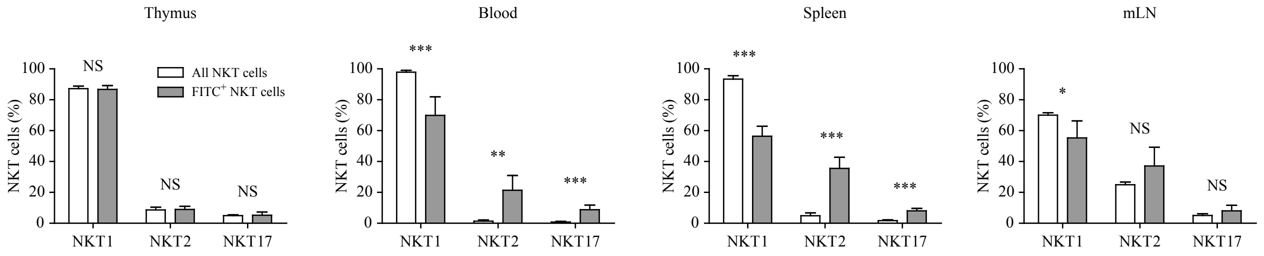

Supplementary Figure 4. Analysis of iNKT RTE of B6.
Mice were injected with FITC intra-thymically and the RTE (DAPI$^-$B220$^-$tet$^+$) analyzed 40h later. Shown is the iNKT subtype composition in all iNKT cells (open bars) in the compartment indicated or only that of iNKT RTE (FITC$^+$, grey bars). Shown are means ± SD (n=5), data pooled from 2 independent experiments. An unpaired two-tailed t-test was performed. NS, not significant ($P > 0.05$), $*P < 0.05$, $**P < 0.01$ and $***P < 0.001$.

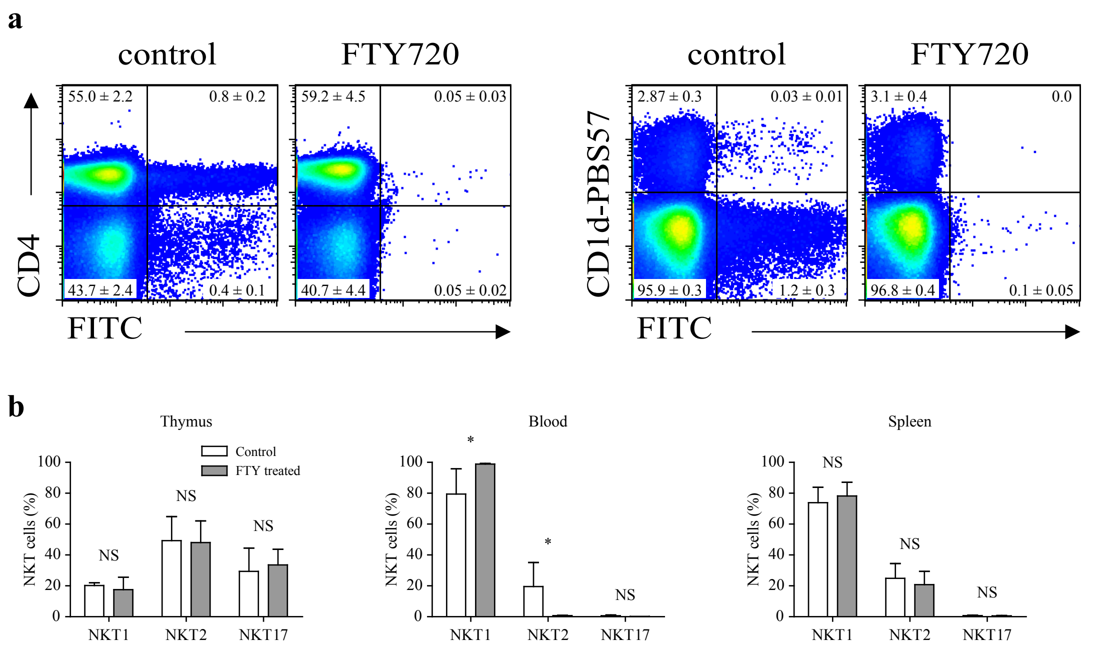

Supplementary Figure 5. Effects of FTY720 treatment on iNKT subset composition.
(a) Frequencies of RTE ($FITC^{+}$) are drastically reduced among spleen cells ($DAPI^{-}B220^{-}$) of FTY720 treated animals. Given are the percentages of cells (means ± SD, n=3 control, n=5 FTY720) when gated for CD4 (left panels) or for tet (right panels). **(b)** Summary of data shown as exemplary plots in Fig. 5g. Open bars: control; grey bars: FTY720 treated. Shown are means ± SD (n=3-5), data pooled from 2 independent experiments. An unpaired two-tailed Mann-Whitney test was performed. NS, not significant ($P > 0.05$) and $^{*}P < 0.05$.

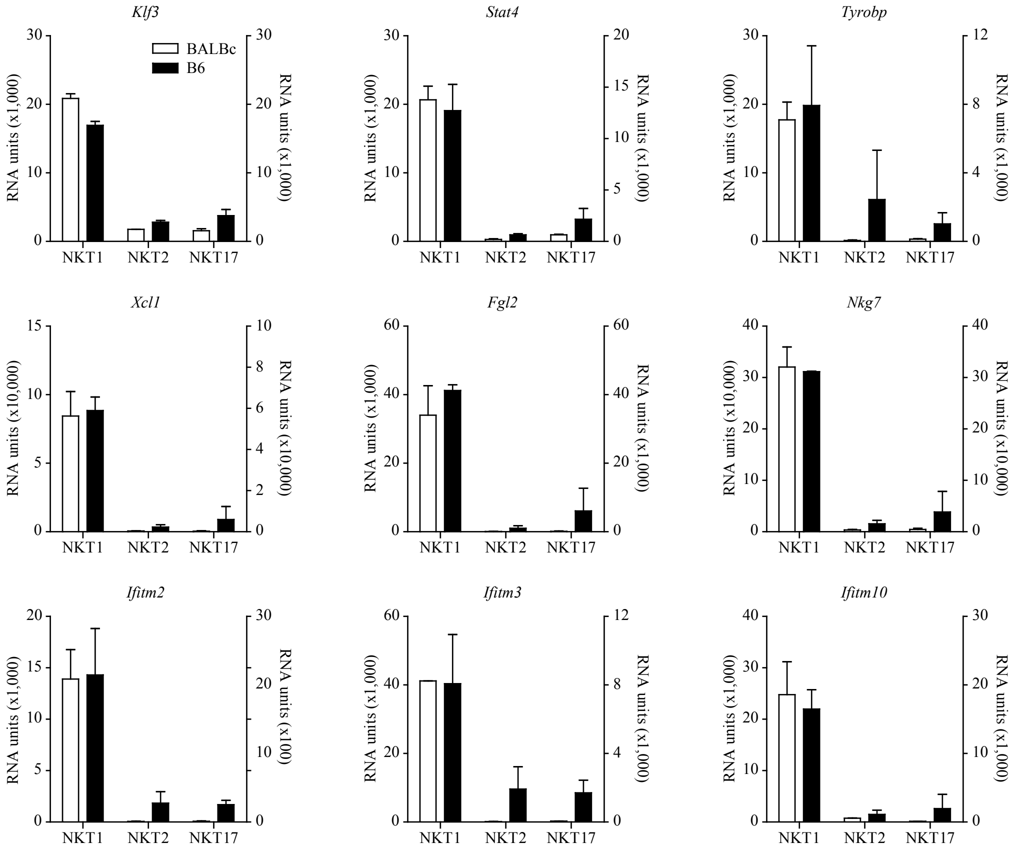

Supplementary Figure 6. Expression of NK-relevant genes by iNKT cells: a selection. Shown are results from transcriptome analyses as presented in Fig. 1e,f. Open bars: BALB/c, black bars: B6; shown are means ± SD.

<u>Supplementary Table 1.</u> BALB/c vs B6: Differently expressed genes by iNKT cell subsets, a selection.

<u>Note:</u> A count of 20 units indicates absence of a detectable signal. See also Online Methods. Values of two independent experiments are listed separately for each strain.

		**BALB/c**			**B6**		
**Gene**	**Protein**	**iNKT1**	**iNKT2**	**iNKT17**	**iNKT1**	**iNKT2**	**iNKT17**
**Expression uniformly higher in B6 than in BALB/c**							
Trim12a	TRIM12A	95<br>54	52<br>46	20<br>24	25,589<br>24,305	16,642<br>16,626	17,165<br>13,810
FJ654066	Endog. retrovirus	20<br>20	20<br>20	20<br>20	24,787<br>16,538	23,809<br>18,912	15,437<br>11,670
Vps16	HVPS16	230<br>439	126<br>252	240<br>270	9,726<br>10,062	7,883<br>8,565	7,382<br>10,074
Ly6a	Ly-6A.2	3,920<br>4,166	2.776<br>1.947	1.784<br>1.068	69,118<br>73,084	71,927<br>72,954	106,158<br>120,228
Ly6e	Ly-6E	20,298<br>38,749	30,368<br>59,990	33,955<br>57,076	245,528<br>236,946	213,131<br>197,119	219,698<br>191,734
**Expression uniformly higher in BALB/c than in B6**							
Scg5	7B2	20,675<br>17,556	9,426<br>7,373	33.939<br>26.414	357<br>436	57<br>119	219<br>308
Ctsl	Cat L	13,005<br>21,006	18,889<br>16,949	6.812<br>5.787	807<br>1,307	627<br>1,182	1,479<br>2,626
Pianp	PANP	3,531<br>4,518	2,403<br>3,303	2.735<br>3.002	20<br>20	20<br>20	20<br>20
Sfi1	SFI1 homolog	3,992<br>4,463	3,273<br>3,172	4.692<br>3.397	119<br>165	125<br>100	82<br>111
**Biased expression on iNKT subtypes + different between BALB/c and B6**							
Klrb1c	NK1.1	2,475<br>2,565	416<br>248	3,985<br>3,081	51,418<br>71,400	3,507<br>3,017	21,097<br>15,069
Klrb1a	NKR-P1A	1,106<br>753	1,544<br>1,030	1,135<br>789	20,272<br>23,529	2,394<br>1,880	9,213<br>4,920
Klri2	KLRI2	179<br>256	20<br>20	20<br>20	3,602<br>5,260	27<br>20	454<br>62
Ncr1	NKp46	7,176<br>5,398	24<br>20	22<br>20	74<br>41	20<br>20	20<br>20
Crtam	CD355	1,800<br>1,643	3,279<br>1,672	23,489<br>10,102	270<br>222	414<br>549	1,258<br>1,990
Ccr5	CCR-5	4,664<br>2,350	182<br>43	102<br>88	545<br>733	124<br>163	214<br>76
Hey1	HESR-1	195<br>170	12,154<br>12,287	1,058<br>1.20	77<br>77	697<br>875	318<br>461
Cd2	CD2	64,038<br>63,725	9,514<br>8,523	1,413<br>2,626	65,697<br>69,453	31,737<br>37,944	28,033<br>16,667
Deptor	DEPDC6	20<br>72	188<br>111	2,325<br>2,183	47<br>35	74<br>90	215<br>400
Mtcl1	SOGA2	20<br>63	89<br>140	3,764<br>4,209	20<br>20	20<br>20	326<br>626
Ascl1	ASH1	59<br>77	164<br>145	6,450<br>2,681	22<br>54	20<br>42	76<br>144
Coro2a	IR10	22<br>20	20<br>20	20<br>20	5,629<br>4,745	894<br>384	2,097<br>979
Car2	CA-II	5,419<br>4,224	224<br>240	209<br>172	234<br>215	36<br>68	40<br>38
Nsg2	Protein p19	9,812<br>7,416	1,553<br>2,052	141<br>203	4,247<br>4,740	23,537<br>23,842	10,936<br>10,067

Supplementary Table 2. Transcriptome data for selected genes BALB/c.

Note: A count of 20 units indicates absence of a detectable signal. See also Online Methods. Genes displaying a > 4 fold preferential expression in either subset over both others are colored: iNKT1 = red; iNKT2 = green; iNKT17 = blue.

**Interleukins**[1]		Experiment 1			Experiment 2		
**Gene**	**Protein**	**iNKT1**	**iNKT2**	**iNKT17**	**iNKT1**	**iNKT2**	**iNKT17**
Il2	IL-2	732	21	20	674	20	20
Il4	IL-4	1,788	39,186	11,378	2,585	58,887	9,298
Il13	IL-13	78	4,640	179	159	6,002	164
Txlna	IL-14	3,990	4,124	4,077	3,734	4,170	4,026
Il16	IL-16	12,414	7,793	12,965	15,039	10,982	15,641
Il17a	Il-17A	20	20	262	20	20	156
Il21	IL-21	34	1,057	146	21	1,332	287
Ifng	IFNγ	20,682	182	482	20,096	212	81
**Interleukin receptors**[2]		Experiment 1			Experiment 2		
Il1r1	CD121a	104	726	24,244	282	983	21,451
Il2ra	CD25	869	199	3,500	708	155	2,368
Il2rb	CD122	147,275	8,923	10,117	131,762	13,208	13,050
Il2rg	CD132	52,328	42,354	33,649	60,804	48,841	49,848
Il6ra	CD126	1,671	28,638	5,052	1,309	26,746	6,327
Il7r	CD127	105,742	66,173	139,653	86,156	49,130	109,523
Il10ra	CD210	7,551	29	34	6,431	132	99
Il17rb	IL-17RB	181	3,954	2,633	207	4,316	3,769
Il17re	IL-17RE	215	684	22,507	475	874	25,282
Il23r	IL-23R	20	532	17,872	275	503	14,613
Ifngr1	CD119	92,849	9,769	66,060	95,438	9,327	41,229
**Chemokines**[3]		Experiment 1			Experiment 2		
Ccl4	MIP-1β	7,063	7,732	6,314	6,947	5,669	5,419
Ccl5	RANTES	269,939	412	278	451,315	410	1,670
Ccl6	MRP-1	2,997	210	547	1,638	20	114
Cxcl11	ITAC	306,158	249,622	323,140	484,549	379,004	442,138
Xcl1	LTN	97,081	376	206	71,881	637	692
**Chemokine receptors**[4]		Experiment 1			Experiment 2		
Ccr2	CD192	3,677	2,237	9,726	2,557	1,765	5,674
Ccr4	CD194	323	6,784	2,626	300	4,378	1,283
Ccr5	CD195	4,664	182	102	2,350	43	88
Ccr6	CD196	107	148	7,555	886	204	12,381
Ccr7	CD197	6,331	37,384	6,445	6,584	27,391	6,974
Ccr9	CDw199	4,499	7,858	861	4,362	7,370	1,268
Ccr10	CCR-10	3,176	26,079	19,308	1,799	12,249	14,359
Cxcr3	CD183	122,829	14,613	1,840	100,084	9,981	1,577
Cxcr6	CD186	223,300	83,593	221,076	182,366	68,151	173,615

**Integrins**[5]		Experiment 1			Experiment 2		
**Gene**	**Protein**	**iNKT1**	**iNKT2**	**iNKT17**	**iNKT1**	**iNKT2**	**iNKT17**
Itga1	α1-chain	3,258	20	20	2,184	20	20
Itga4	α4-chain	9,595	1,602	182	7,786	1,224	365
Itga6	α6-chain	5,236	5,316	8,316	3,129	4,248	6,205
Itgam	αM-chain /CD11b	26,329	11,557	17,761	55,562	29,627	35,007
Itgae	αE-chain /CD103	8,507	11,077	17,450	6,219	5,655	16,021
Itgal	αL-chain /CD11a	10,003	9,029	10,343	8,096	9,790	10,951
Itgb1	β1-chain	10,804	4,043	2,067	8,885	1,955	1,244
Itgb2	β2-chain	44,437	25,123	14,970	36,915	28,617	16,859
Itgb3	β3-chain	4,286	40,676	55,610	2,265	33,549	40,542
Itgb7	β7-chain	54,399	24,875	67,558	48,804	24,439	73,464
**Toll like receptors**[6]		Experiment 1			Experiment 2		
Tlr1	CD281	4,349	8,271	9,518	3,807	8,963	9,542
Tlr6	CD286	811	789	929	579	652	568
Tlr7	TLR7	3,028	4,171	2,821	7,264	4,897	5,945
Tlr9	CD289	2,326	1,677	2,029	2,321	1,927	2,575
Tlr12	TLR12	1,578	741	234	1,357	638	386
**Transcription/ Nuclear factors**[2]		Experiment 1			Experiment 2		
Zbtb16	PLZF	8,340	44,013	40,517	8,765	54,910	34,890
Tbx21	T-bet	39,615	1,282	218	36,849	1,153	416
Rorc[7]	RORγ,γt	88	307	5,394	294	438	5,860
Gata3	GATA-3	21,778	18,009	27,855	13,917	15,949	14,211
Zbtb7b	THPOK	12,345	15,722	15,178	18,022	20,816	19,819
Egr2	EGR2	2,357	5,205	3,576	2,209	6,288	2,942
Batf	B-ATF	8,661	9,657	17,049	8,810	9,362	18,099
Vdr	VDR	233	2,407	6,416	149	2,846	3,224
Klf3	BKLF	20,394	1,744	1,784	21,334	1,769	1,362
Stat4	STAT4	22,074	223	1,038	19,243	362	892
Tcf12	HEB	9,385	7,319	6,684	9,508	6,615	5,907
Id2	BHLHB26	149,554	136,951	146,254	156,747	145,636	118,625
Id3	BHLHB25	18,584	142,240	143,248	17,212	170,789	83,048
Lef1	LEF-1	1,658	6,589	1,533	1,561	6,459	1,560
**Cell signaling/ Apoptosis**[2]		Experiment 1			Experiment 2		
Hcst	DAP10	150,118	71,676	94,293	135,201	64,619	60,822
Tyrobp	DAP12	19,575	194	383	15,880	65	300
Mapk10	MAPK10	72	936	28,793	162	263	8,295
Blk	BLK	222	920	34,986	341	792	30,431
Dapk2	DAPK2	11,256	653	110	10,693	527	130
Socs2	SOCS2	5,321	166	2,846	3,912	115	1,996
Socs3	SOCS3	3,928	1,835	33,758	4,064	3,028	32,620
Bcl2	BCL2	6,631	478	508	3,923	630	466
Faslg	CD95L	19,327	447	131	15,990	573	227

1) Interleukins including IFNγ; mRNAs giving rise to less than 150 units in all subpopulations are neglected, otherwise all available data for interleukins are displayed.
2) Only a selection of expressed genes is displayed.
3) Chemokine mRNAs giving rise to less than 1,000 units in all subpopulations are neglected, otherwise all available data for chemokines are displayed.
4) Chemokine receptor mRNAs giving rise to less than 1,000 units in all subpopulations are neglected, otherwise all available data for chemokine receptors are displayed.
5) Integrin chain mRNAs giving rise to less than 1,500 units in all subpopulations are neglected, otherwise all available data for integrins are displayed.
6) TLR mRNAs giving rise to less than 500 units in all subpopulations are neglected, otherwise all available data for TLR are displayed.
7) The probe used to detect *Rorc* transcription cannot discriminate whether RORγ or RORγt will be produced.
